# Biogenesis, Trafficking, and Function of Small RNAs in Plants

**DOI:** 10.3389/fpls.2022.825477

**Published:** 2022-02-17

**Authors:** Yunjia Tang, Xiaoning Yan, Chenxian Gu, Xiaofeng Yuan

**Affiliations:** ^1^College of Life Science, Zhejiang Chinese Medical University, Hangzhou, China; ^2^Academy of Chinese Medical Sciences, Zhejiang Chinese Medical University, Hangzhou, China

**Keywords:** small RNA, biogenesis, trafficking, functions, DNA methylation, RNA interference, translational repression

## Abstract

Small RNAs (sRNAs) encoded by plant genomes have received widespread attention because they can affect multiple biological processes. Different sRNAs that are synthesized in plant cells can move throughout the plants, transport to plant pathogens *via* extracellular vesicles (EVs), and transfer to mammals *via* food. Small RNAs function at the target sites through DNA methylation, RNA interference, and translational repression. In this article, we reviewed the systematic processes of sRNA biogenesis, trafficking, and the underlying mechanisms of its functions.

## Introduction

Excessive use of pesticides and chemical fertilizers has already caused dramatic damages to the ecological environment, yet some pests and plant diseases are still not completely controlled and prevented. Thus, extensive attentions have recently been paid on biological control approaches (Ma et al., 2021; Peng et al., 2021). Biological control approaches are highly effective to prevent plant diseases and increase the crop yields. In most eukaryotes, small RNAs (sRNAs) are generated by the ribonuclease III-like enzyme dicer or dicer-like (DCL) proteins and are incorporated into argonaute (AGO) proteins to induce gene silencing in a sequence-specific manner (Huang et al., 2019a). Small RNAs are widely present in plants and have been gradually utilized to control plant diseases and insect pests because they can regulate various biological processes, e.g., plant growth, development, and stress response (Si et al., 2020; Mekapogu et al., 2021).

To identify functional sRNAs, it is necessary to construct an sRNA library which is often completed by sRNA sequencing, DNA microarray, and shotgun cloning (Vogel et al., 2003; Boccara et al., 2017; Wu et al., 2020). The presence of sRNA in plants can be verified by RNA blotting, quantitative PCR and other techniques (Cai et al., 2018; Cui et al., 2020). Dual-luciferase reporter assay, gene transient expression analysis, and degradome sequencing are usually used to verify sRNA binding sites (Hu et al., 2020; Xie et al., 2021). The function of sRNAs can be analyzed by constructing transgenic plants using short tandem target mimic (STTM), CRISPR-Cas9, homologous recombination and other gene editing technologies (Cui et al., 2020; Qiao et al., 2020; Ji et al., 2021). To better understand the function of sRNA and promote the use of sRNA in agricultural production, we reviewed the process of sRNA biogenesis, trafficking and the underlying mechanisms of its functions.

## sRNA biogenesis

Plant sRNAs are generally divided into two main categories, microRNAs (miRNAs) and small interfering RNAs (siRNAs). In plants, siRNAs can be generated through multiple biogenesis pathways (Borges and Martienssen, 2015). However, the pathway for miRNA biogenesis is unique. Based on the biogenesis and biosynthesis, siRNAs can be further divided into natural antisense transcript small interfering RNA (natsiRNA), heterochromatic small interfering RNA (hcsiRNA), virus-derived small interfering RNA (vsiRNA) and secondary siRNA (Song et al., 2019; Zhang et al., 2019; Middleton et al., 2021).

### Biogenesis of miRNA

**Figure 1:**
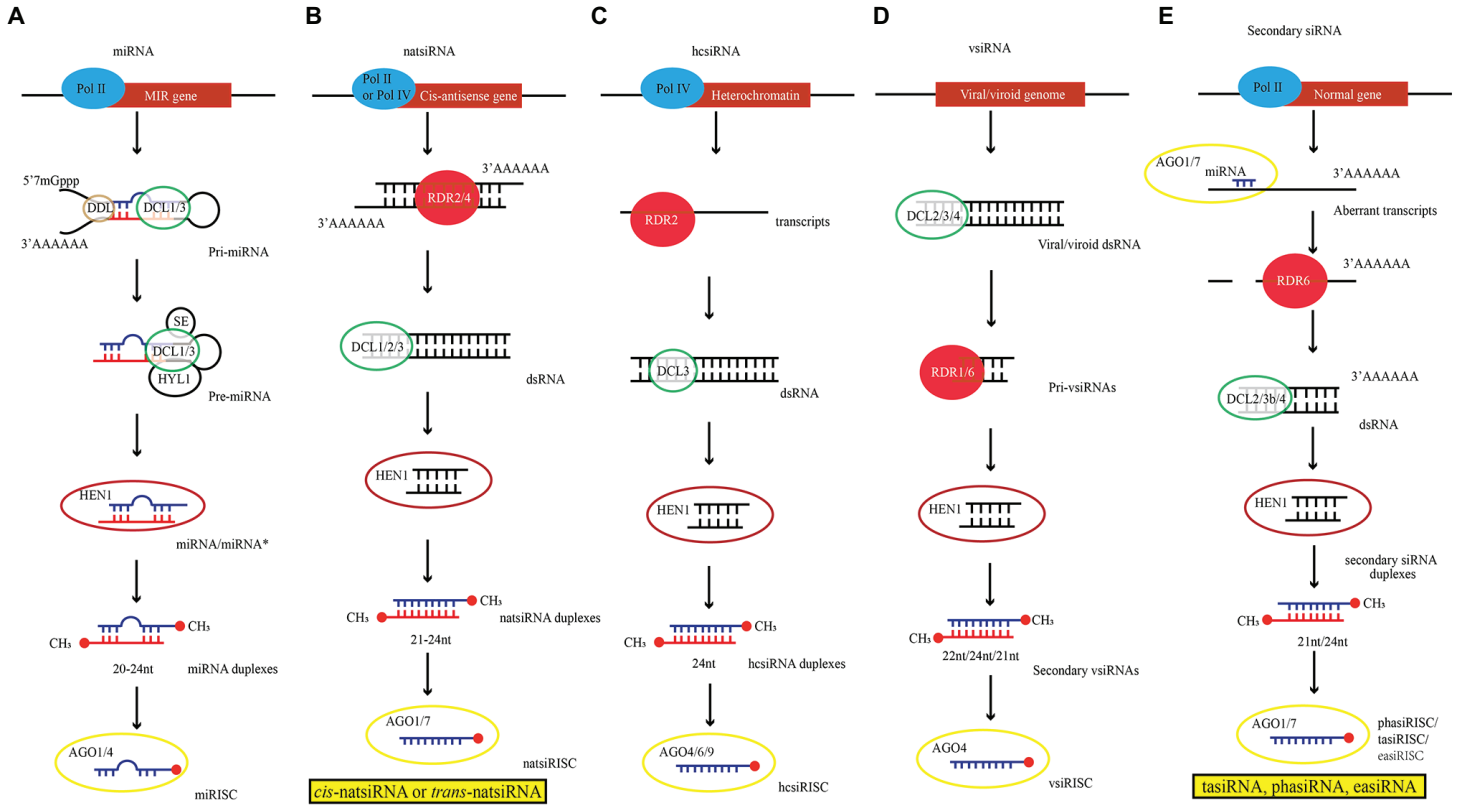
Illustrations of siRNA biogenesis in plants (Ref Borges et al., 2018; Singh et al., 2021). **(A)** miRNA biogenesis model derived from MIR gene. **(B)** natsiRNA biogenesis model derived from Cis-antisense gene. **(C)** hcsiRNA biogenesis model derived from Heterochromatin. **(D)** vsiRNA biogenesis model derived from Viral/viroid genome. **(E)** Secondary siRNA biogenesis model derived from Normal gene. Pol: RNA Polymerase; DDL: Dawdle; DCL: RNase III enzyme DICER-LIKE; SE: SERRATE; HYL1: HYPONASTIC LEAVES1; HEN1: HUA ENHANCER 1; RDR: RNA-DEPENDENT RNA POLYMERASE.

Transcription of miRNA genes (MIRs) in euchromatic regions of plant chromosomes is catalyzed by DNA-dependent RNA Polymerase II (Pol II; Figure 1A; Xie et al., 2005). The primary transcript of miRNA (pri-miRNAs) contains at least one characteristic hairpin-like structure. Subsequently, pri-miRNAs are loaded into nuclear dicing bodies (D-bodies) including DCL1, HYPONASTIC LEAVES 1 (HYL1), SERRATE (SE) and TOUGH (TGH; Fang and Spector, 2007). Then, DCL1 cuts the hairpin structure on the pri-miRNA through two consecutive cleavage steps, resulting in a miRNA duplex of approximately 21 nucleotides (nt; Kurihara and Watanabe, 2004). Following pri-miRNA processing, HUA ENHANCER 1 (HEN1) catalyzes 2’-O-methylation at the 3′-ends of miRNA duplex so that miRNAs are more stable (Huang et al., 2009). This mature miRNA duplexes are loaded into AGO1 protein and form an miRNA-induced silencing complex (miRISC) with the assistance of heat shock protein (HSP70/HSP90) and Constitutive Alterations in the Small RNAs Pathways9 (CARP9; Bologna et al., 2018; Tomassi et al., 2020). In RISC, only one strand from the miRNA duplex was usually loaded, and the other strand with higher thermodynamic stability at the 5′-end was degraded. For miRNA/miRNA* duplex, the miRNA* strand was usually degraded. However, miRNA* strand can also be accumulated and loaded into AGO protein (Eamens et al., 2009; Song et al., 2019). In addition, other models also propose that DCL3 can produce 24-nt miRNA. The trafficking and function of 24-nt miRNA are different from those of 21-nt miRNA (Cervantes-Pérez et al., 2021).

In general, MIR gene is not static and can evolve with the changing environment. Because MIR gene is evolving, the lineage-specific miRNAs between species are created and may guide the co-evolution of mRNA target sequences (Cui et al., 2017). Biogenesis of miRNAs can be regulated by both transcriptional and post-transcriptional factors (Voinnet, 2009; Li and Yu, 2021). For instance, 3′-phosphoadenosine 5′-phosphate (PAP) and Tocopherols (vitamin E) can protect pri-miRNAs from being degraded and promote the production of mature miRNA in *Arabidopsis thaliana* (Fang et al., 2019). RNA adenosine methylase (MTA) catalyzes the formation m6A on pri-miRNAs to modulate miRNA biogenesis (Bhat et al., 2020). The abundance of miR156 is positively regulated by AGL15 because AGL15 can inhibit the expression of DCL1 and SERRATE genes (Nowak et al., 2020). Nucleoplasmic exosome protein is an RNA processing complex containing 3′-5’exoribonuclease. HYL1 can promote pri-miRNA processing and prevent the attack from exosome (Gao et al., 2020). Gene cma33/XCT can regulate sRNA biogenesis through controlling the transcription of DCL gene (Fang et al., 2015).

Biosynthesis of miRNA is regulated not only by genetic factors under normal conditions but also by environmental factors, e.g., changes in the environmental stress. For example, strontium stress inhibits the biogenesis of miRNA by reducing the level of HYL1 protein in *Arabidopsis* (Pyo et al., 2020). Under environmental stress, MPK3 and SnRK2 can phosphorylate and inactivate cofactors, e.g., HYL1 and SE, leading to the decreases in the production of miRNA (Manavella et al., 2019). The activity of mitochondria is greatly inhibited by hypoxia condition, which triggers the biogenesis of miRNAs responsible for hypoxia tolerance (Betti et al., 2020). Under the environmental stress, plants can make corresponding adjustments *via* regulating the biosynthesis of miRNAs to maintain their own life activities.

### Biogenesis of siRNA

In general, dsRNAs that are the precursors of siRNAs are produced through two different pathways. The first is from the abnormal transcripts of genes (including the hybridization of sense and antisense transcripts, the folding back of an inverted-repeat sequence, and the hybridization of unrelated RNA molecules with sequence complementarity) that are subsequently processed and loaded by RDRs and SGS3 (Marchais et al., 2019), and the second is from single-stranded RNA after being processed and loaded by RNA polymerase IV (Pol IV) and RDRs. The dsRNA formed by these two pathways is processed by DCL2/3/4 into 21 ~ 24 nt siRNA (Yao et al., 2020).

Natural antisense transcripts (NATs) are formed by annealing of two complementary and separately transcribed RNA strands. According to their genomic origin, they can be divided into cis-NAT and trans-NAT. Cis-NAT is transcribed from the same genomic locus, forming a completely complementary dsRNA between the two transcript sequences. In contrast, trans-NAT constitutes highly complementary dsRNAs encoded by two distant genomic sites. Amplification of these two dsRNAs requires the participation of RDR2/4(Yu et al., 2016). Subsequently, with the participation of cofactors, e.g., RDR6, SGS3, and DNA directed RNA polymerase IV subunit 1 (NRPD1), DCL1/2/3 cleave natsiRNA precursor, leading to the production of 21 ~ 24 nt *cis*-natsiRNA or *trans*-natsiRNA (Zhang et al., 2012; Figure 1B). The hcsiRNA is derived from repetitive sequences on chromatin and transposable elements (TE). Its biogenesis also requires RNA Pol IV-mediated transcription and RDR2-mediated formation of dsRNA (Parent et al., 2015). Finally, DCL3 processes the dsRNA into 24 nt siRNA duplexes, and HEN1 methylates the siRNA duplexes to form 24 nt hcsiRNA (Chen et al., 2021a; Figure 1C). Biosynthesis of vsiRNA also requires the participation of DCLs, AGOs, and RDR proteins. The difference is that vsiRNA originates from abnormal transgene (produced by viral DNA) or viral RNA in plants after virus infection. RNA-dependent RNA polymerase (RdRP) may recognize and use these abnormal RNAs as templates to synthesize antisense RNA and form dsRNA (Leonetti et al., 2020). These dsRNAs are processed by DCL2/3/4 to produce 22, 24, and 21 nt primary vsiRNAs, respectively, which are subsequently amplified by RDRs and loaded into AGOs to form vsiRNA (Garcia-Ruiz et al., 2010; Vivek et al., 2020; Figure 1D).

PolII catalyzes the transcription of plant genes (including PHAS loci, TAS gene, and active retrotransposons). After the transcripts are cleaved by sRNA, the 5′ end fragment of the transcript is degraded, while the 3′ end fragment is converted into dsRNA by RDR6 and becomes the precursor of secondary siRNA. Subsequently, dsRNA is processed by DCL2/4 to generate 21 ~ 24 nt siRNA (Figure 1E). They are subdivided into phased siRNA (phasiRNA), trans-acting siRNAs (tasiRNA) and epigenetically activated siRNAs (easiRNA). All the three subclasses of siRNAs are generated *via* different biogenetic pathways. For example, there are two mechanisms for the biogenesis of phasiRNA: “one-hit” and “two-hit” modes (Liu et al., 2020). In the “one-hit” mode, the 22 nt miRNA cleaves the mRNA from the 3′ end at the single target site to generate phasiRNAs with the participation of factors, e.g., RDR6, DCL4, and DCL3b (Tian et al., 2021). However, in the “two-hit” mode, although mRNA contains two miRNA target sites, only one site can be cleaved (usually at the 3’end site), and mRNA is cleaved successively by DCL4 to produce 21 nt phasiRNA (Axtell et al., 2006). As one of phasiRNAs, tasiRNA is produced by miRNA-guided cleavage of long and noncoding precursor transcripts. The cleaved fragments are then converted to dsRNAs by RDR6 and processed into 21 nt siRNAs by DCL4 (Yang et al., 2021b). Moreover, easiRNA is originated from active transposons in plants and is also produced by DCL2/4 processing (Creasey et al., 2014; Wu et al., 2020). SGS3 mediates the specific recognition of RDR6, which specifically recognizes the transposon RNA and synthesizes dsRNA (Kim et al., 2021).

The biosynthesis of siRNA and miRNA is also regulated by environmental factors. AGO1 could accumulate miRNA into the membrane-bound polysomes (MBPs) and cleave the targeted transcripts to produce phasiRNA. In other words, under specific condition, AGO1 affects the synthesis of phasiRNAs by regulating the membrane binding of miRNAs (Li et al., 2016; Komiya, 2017). NOT1, as a component of CCR4-NOT complex, regulates DNA methylation and transcriptional silencing by promoting the production of Pol-IV-dependent siRNA (Zhou et al., 2020). Under stress conditions, plants preferentially accumulate 22 nt siRNA from NIA1/2 gene to inhibit plant growth and enhance stress response (Wu et al., 2020). Thus, biogenesis of siRNA in plants is also regulated to ensure its rational synthesis.

### Biogenesis of Other sRNAs

miRNA and siRNA are the two common sRNAs in plants. However, to meet additional regulations, other sRNAs are also generated. For example, tRNA-derived RNA fragments (tRFs) are generated by excising from mature tRNA or produced as a by-product of pre-tRNA processing (Megel et al., 2019). According to the cleavage sites, tRF can be divided into tRF-5a and tRF-3a (Park and Kim, 2018). At present, the biogenesis of tRFs in plants is not clear. However, it is known that tRFs are also loaded onto AGO1/2/4, indicating that rRFs execute their functions of gene silencing similarly as miRNA and siRNA (Ren et al., 2019).

siRNAs independent of DCLs (sidRNAs) is considered as a new type of sRNA, which is mainly originated from the sidRNA loci on transposons, intergenic sequences or transgenes (Ye et al., 2016). The sidRNA loci are transcribed to form precursors under the action of PolIV and RDR2. Subsequently, 24 nt sidRNA is produced by 3′-5′ exonuclease, and gene *Atrimmer* may be a potential splicing site. SideRNAs recruits AGO4 to the target sites, implying similar mode with other sRNAs (Ye et al., 2016).

The sRNA produced by the plant factory is either transported to subcellular areas, or exposed to ZSWIM8 ubiquitin ligase in the cytoplasm, and then degraded after the 5′ end cap structure is removed by RDR6 (Baeg et al., 2017; Han et al., 2020).

## sRNA Trafficking

One of the most fascinating aspects of sRNA is its mobility, in other words, its ability to spread from one cell to its neighboring cells (Liu and Chen, 2018). In early studies, transfer pathway of sRNA in plants was described as “particle bombardment with siRNA/transgenics” (Agrawal et al., 2003). The logistics network of sRNA has been recently elucidated with the development of various sRNA tracing technologies (Molnar et al., 2010; Huang et al., 2019b). In addition to the trafficking of sRNA within plants, external movements of sRNA have also been observed in plants (Cai et al., 2021).

### sRNA Trafficking Inside Plants

**Figure 2:**
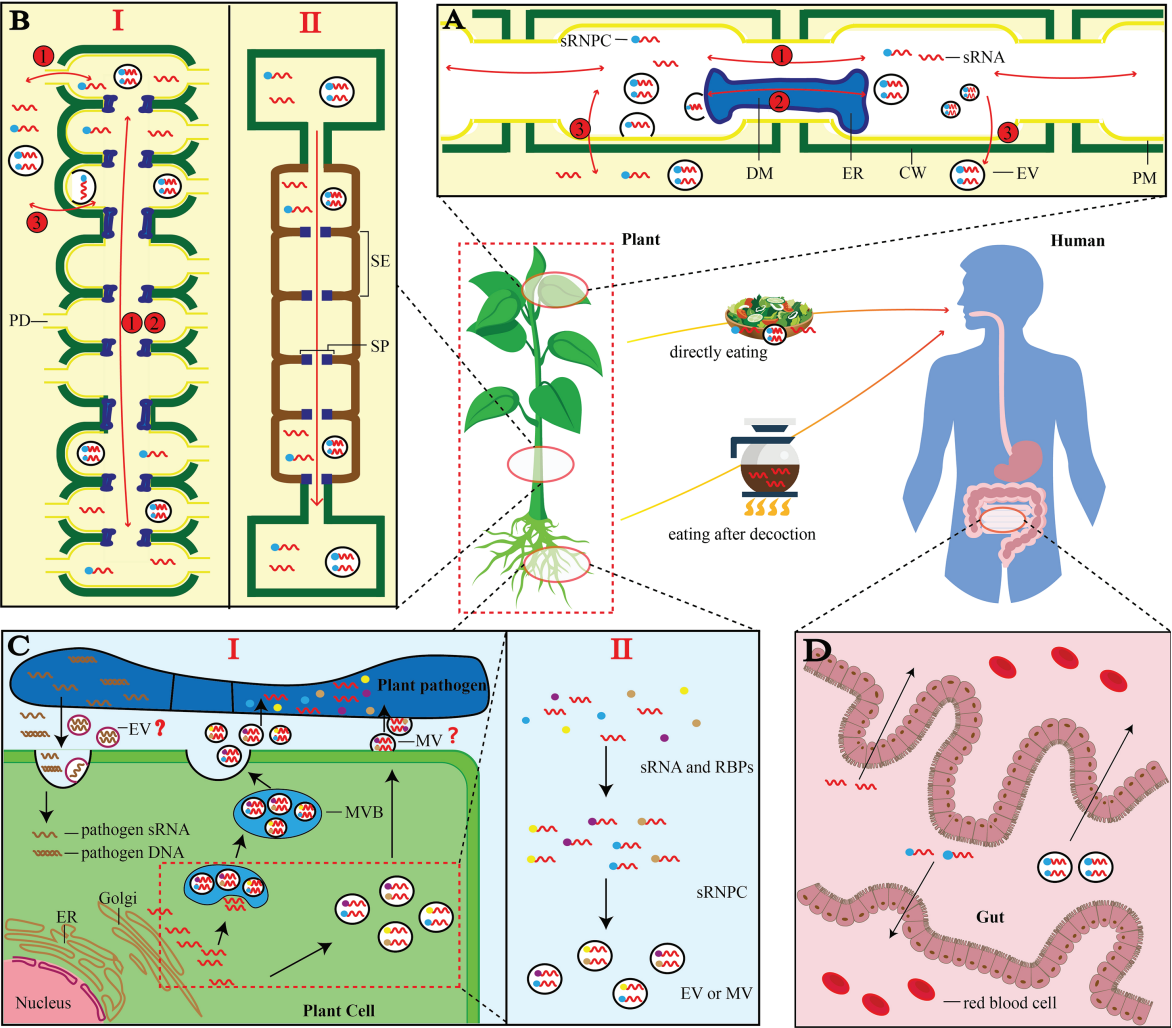
Illustrations of siRNA trafficking in plants. **(A)** Movement between plant cells (Wang and Dean, 2020): Pathway 1, naked small RNAs, small RNAs bound to RNA binding protein (RBP) and small RNAs enclosed in vesicles can pass through plasmodesmata (PD) moves between cells; pathway 2, desmotubules (DM) connects the endoplasmic reticulum (ER) of two adjacent cells, and small RNA can be transported through DM; pathway 3, small RNA can be directly secreted from PM and spread in plants. Note: It is unknown whether vesicles can be transported *via* DM. **(B)** Long-distance movement in plants (Tamiru et al., 2018): I: Long-distance movement occurs through the repetitive mechanism of pathways 1 and 2 in the plasmodesmata; II: sRNA enters the phloem sieve tube through the plasmodesmata (PD), and transports it quickly from top to bottom with the phloem sap. **(C)** Cross-domain transport of sRNA by EV (Huang et al., 2019a): I: Free sRNA in plant cytoplasm can be packaged by Golgi and transported to the outside of the cell to be absorbed by Plant pathogen. At the same time, Plant pathogen also produces sRNA and delivers its own DNA or sRNA to plant cells; II: When vesicles are formed, sRNA needs to be combined with RBPs before it can be selectively loaded into EVs for cross-domain transport. Note: Plant pathogen here is only a type of organism that absorbs vesicles. Many organisms can absorb vesicles provided by plants or produce sRNA and transfer them to plant cells. “?” indicates whether plants can produce MV and whether other organisms can produce EVs is not yet clear; **(D)** Transfer of sRNA to animal cells: Plants deliver sRNA to the human body through decoction or raw food directly. After different forms of sRNA enter the gut, it is absorbed into the blood through the villi of the small intestine and circulates throughout the body with the blood. Human here refers to mammals. sRNA: small RNA; MVB: multivesicular bodies; sRNPC: sRNA ribonucleoprotein complex; DM: desmotubule; ER: endoplasmic reticulum; CW: cell wall; EV: extracellular vesicles; PM: plasma membrane; PD: plasmodesmata; SE: sieve tube elements; SP: sieve tube plates; MV: microvesicles.

After sRNA is synthesized in the cell nucleus, it is loaded into the AGO protein. Then, the nuclear localization signal and nuclear export signal (NES) directly guide the nucleocytoplasmic shuttle of RISC (Bologna et al., 2018). Subsequently, there are three main forms of sRNA involved in transfer: naked sRNAs, sRNAs bound to RNA-binding proteins (RBPs) and sRNAs inside vesicles (Wang and Dean, 2020). Short-range movement between cells occurs through plasmodesmata (PD; Garnelo Gómez et al., 2021). PD is a membrane channel that passes through the cell wall and connects adjacent cells through the plasma membrane (PM). In the channel, there is a specialized-cylindrical structure called desmotubule (DM). DM is derived from the smooth endoplasmic reticulum and can connect the endoplasmic reticulum of two cells. sRNA could move between cells through PD pore or along the desmosomes (Di Donato and Amari, 2014; Figure 2A, pathway 1, 2). The dominant sRNA involved in short distance movement is the 21-nt sRNA (Tamiru et al., 2018). SUC-SUL and SUC-PDS are artificial siRNA reporter systems. In both systems, long inverted-repeat dsRNAs are expressed in phloem companion cells. siRNA can be produced and diffused to 10–15 neighboring cells, reflecting its local cell-to-cell movement (Liu and Chen, 2018). It is worth noting that sRNA can bind RBPs to form sRNA ribonucleoprotein complexes (sRNPC), which are co-transported between cells. A recent study identified a conserved RBP (SRBP1) in the phloem of cucurbit, which mediates the trafficking of siRNA between cells (Yan et al., 2020). Therefore, detailed information on RBPs would facilitate the understanding on the regulation of sRNA movement. In addition to the mainstream PD transport pathway, both naked sRNAs and sRNAs inside vesicles can be secreted directly from PM and spread between plant cells (Weiberg et al., 2013; Cai et al., 2019; Figure 2A, pathway 3).

The most intuitive model for the long-distance movement of sRNA is the molecular signal transmission model between scions and rootstocks of grafted plants. The transfer of transgene-derived siRNA from rootstock to scion makes non-transgenic cherry scion resistant to the Prunus necrotic ringspot virus. 24-nt sRNA can also be transferred from cherry scion to the rootstock, which potentially affects the rootstock (Zhao and Song, 2014; Zhao et al., 2020). Long-distance root-to-shoot movement occurred intercellularly *via* plasmodesmata by a repeating mechanism (Tamiru et al., 2018). In this mechanism, the three forms of sRNA could still be transported *via* PD or directly across the PM (Figures 2A,B, I). Notably, the permeability of PD is strongly regulated by several factors, e.g., endogenous reactive oxygen species (ROS; Welchen and Gonzalez, 2021), light and circadian clock (Brunkard and Zambryski, 2019).

There is a special pathway in the long-distance transportation of sRNA from the top to the bottom of plants (Figure 2B, II). The phloem protein kinase PSRPK1 is phosphorylated by PSRP1 to form sRNA ribonucleoprotein complex (sRNPC) after sRNA is produced in the cell. sRNPC passes through the PD and enters the phloem sieve tube. During the long-distance movement, PSRP1-sRNPC is stable against the phloem phosphatase activity (Ham et al., 2014). Small RBP-bound RNAs pass through septum (SP) and are rapidly transported to target tissues by phloem sap. Subsequently, they are unloaded into the surrounding cells followed by decomposition of PSRP1-SrNPC complex (Ham et al., 2014). For the transport of sRNAs inside the vesicles, only vesicles are found in the phloem, while sRNA inside the vesicles needs to be further identified and characterized (Chukhchin et al., 2019). The long-distance transportation of sRNA from the bottom to the top of the plant also has a special route based on the xylem catheter, which is not well characterized. Cadmium treatment can alter miRNAs in leaves of maize and xylem sap, indicating that sRNA is involved in the stress response of plants, and xylem catheter can transport these sRNAs (Wang et al., 2019).

### sRNA Is Selectively Packed Into Vesicles

It is initially believed that the outer vesicles are just a way for cells to discharge metabolic waste, but further researches reveal that they are rich in protein and sRNA and could function in a cross-regional or cross-species manner (Thomma and Cook, 2018). The discovery of extracellular vesicles is a breakthrough in the field of secretion, as it provides a new mechanism for releasing components into the extracellular environment (Shao et al., 2018). The most classic study showed that host *Arabidopsis* cells can secrete extracellular vesicles to deliver sRNAs into fungal pathogen *Botrytis cinerea* (Cai et al., 2018). This mechanism has been discovered in plants such as sunflower, tomato and olive, indicating that the precise cross-kingdom targeting transport of plant sRNA is mediated by EV (Prado et al., 2014; Regente et al., 2017; De Palma et al., 2020; Figure 2C, I). In addition, fungi, bacterial and parasitic plants can also transport sRNA or genes into plants as one of the sources of vsiRNA (Shahid et al., 2018; Dunker et al., 2020; Ji et al., 2021; Figures 1D, 2C, I).

In the past, it was not clear if sRNAs in plant EVs are selectively loaded. In mammalian cells, the mechanism of sRNA loading has been revealed. In cancer cell EVs, members of the hnRNP family, as well as other molecules, e.g., YBX1, HUR, and AGO2 are used as RBPs (Fabbiano et al., 2020). In liver cells, SYNCRIP, involved in the exosomal sorting of miRNAs, interacts with specific miRNAs and binds extra-seed sequence (hEXO Motif), which regulates the localization of miRNAs (Santangelo et al., 2016). During autophagy, specific RBPs need to be loaded into extracellular vesicles through LC3 coupling mechanism (Leidal et al., 2020). A recent groundbreaking study demonstrated for the first time that RBPs affect EV loading of sRNA in plants. Several RBPs in the EV of *Arabidopsis* have been identified, including RBPs Ago1, RHs and ANNs. Studies on gene knockout experiments indicate that these RBPs may contribute to sRNA sorting and stabilization (He et al., 2021). These EV-coated Ago1, RH11 and RH37 may contribute to the selective sRNA sorting and stability in EVs. However, ANN1 and ANN2 only stabilize sRNA in EV, indicating that the vesicle transfer pathway of sRNA requires RBPs for selective loading and cross-domain transport (Figure 2C, II).

### Transfer of sRNA to Animals *via* Food

It has been controversial over whether plant-derived sRNA could pass through the mammalian gastrointestinal tract and enter the bloodstream because there are a series of obstacles in the mouth, stomach, large intestine, and small intestine (Dávalos et al., 2019; Figure 2D). Although it is theoretically difficult, it was found in 2012 that MIR168a carried by ginger-derived nanoparticles could travel to the liver after being absorbed from the gastrointestinal tract (Zhuang et al., 2015). Since then, extensive studies on the transfer of sRNA from plants to mammals have been carried out (Mar-Aguilar et al., 2020; Chen et al., 2021b). For example, some studies showed that sRNA from strawberries, blueberries and other plants have significant effect on human health (De Robertis et al., 2020; Alfieri et al., 2021; Perut et al., 2021). However, other studies argued that the cross-kingdom transfer of exogenous sRNAs was insignificant and biologically irrelevant, and the results lacked reproducibility (Mar-Aguilar et al., 2020). The cross-kingdom transfer of sRNA observed in these studies might be due to experimental artifacts and contaminations (Witwer, 2018).

Surprisingly, some high-temperature-resistant sRNAs in some plants especially Chinese medicinal materials can be preserved after decoction and can be absorbed by the intestines to achieve their potential functions. For example, MIR2911 is not significantly degraded after boiling, and can inhibit the expression of Enterovirus 71 (EV71) and VP1 protein *in vitro* and *in vivo* (Zhou et al., 2015; Li et al., 2018). Similarly, the unique miRNAs of *Gastrodia elata* including GAS-mir01 and gas-mir02 are stable during decoction and long-term preservation, and both could target the human A20 gene *in vitro* (Xia et al., 2020). High GC content in the miRNAs might be the reason of high stability after decoction in these studies. Overall, the evidence for the transfer of miRNAs from diet to blood remains inconclusive, and definitive evidence and reproducible findings are needed (Mar-Aguilar et al., 2020).

## sRNA Functions

After sRNAs are biosynthesized and transported, they function at the target site. It functions only when it binds to the target sites based on AGO-guided watson-crick base pairing rules (Fei et al., 2021). miRNA and siRNA can not only mediate transcriptional gene silencing through RNA-directed DNA methylation (RdDM), but also perform post-transcriptional gene silencing through cleavage and translational inhibition without changing the DNA sequence (Borges and Martienssen, 2015).

### sRNA Mediates Transgenerational Epigenetic Inheritance Through DNA Methylation

**Figure 3:**
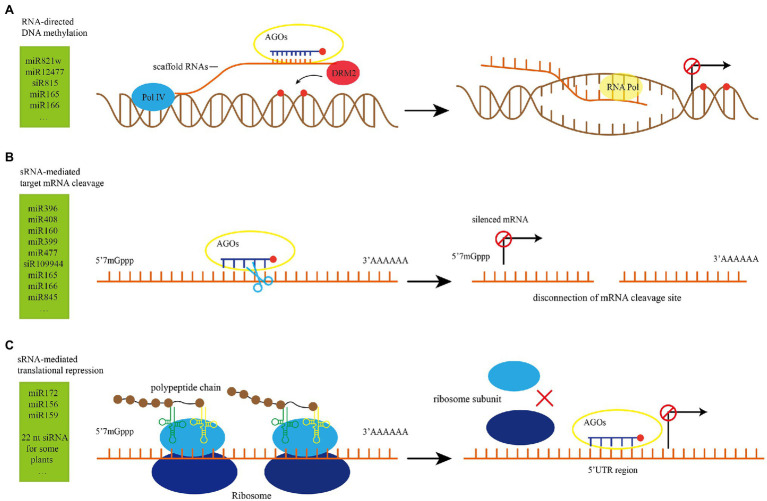
Illustrations of sRNA function. **(A)** RNA-directed DNA methylation (RdDM) model (Tang, 2020). The scaffold RNAs are produced by Pol V, recruit their complementary sRNA to the RdDM target loci and guides DRM2 to catalyze DNA methylation. **(B)** sRNA-mediated target mRNA cleavage model. RISC is paired with target mRNAs according to the principle of base complementary pairing. The PIWI domain of AGO proteins has slicer endonuclease activity, and the paired regions are cleaved with the participation of AGO protein. **(C)** sRNA-mediated translational repression model (Simone et al., 2021): RISC competes with ribosomes for binding to the UTR region of mRNA, which affects the translation process and inhibits the formation of polypeptide chains. RISC: sRNAs carried by RNA-induced silencing complex; UTR: untranslational region; DRM2: DOMAINS REARRANGED METHYLTRANSFERASE 2.

sRNAs that can mediate DNA methylation are derived from the short transcripts of methylated templates (Matzke and Mosher, 2014). Among all kinds of sRNA, 24 nt sRNA is classified as sidRNA (Ye et al., 2016). sRNA can mediate transcriptional gene silencing by RdDM, which includes the initial recruitment of DNA methyltransferase and subsequent catalytic *de novo* DNA methylation of cytosine in all sequences after pairing of AGO loaded siRNA with Pol V transcribed scaffold RNA (Huang et al., 2021; Figure 3A).

RdDM can not only maintain long-term genome stability by inhibiting transposable elements but also ensure plants’ life activities under stress conditions by regulating gene expression (Rymen et al., 2020; Guo et al., 2021). High temperature stress usually promotes RdDM (Singh et al., 2021), which inhibits the expression of ROS1 gene due to hypermethylation and affects seed germination (Malabarba et al., 2021). The slowdown of life activities may be beneficial to tolerate high temperature environments. Moreover, in the chilling environment, sRNA mediates hypermethylation of the dormancy-related gene DAM and initiates dormancy (Zhu et al., 2020a). Meanwhile, Osa-miR12477 regulates the expression of gene LAO to tolerate salt and reduces oxidative damage (Parmar et al., 2020). It has been reported that RdDM occurs in plants in response to abiotic stress environments, e.g., drought and salinization and biological stresses (Erdmann and Picard, 2020; Liu and He, 2020; Kumar and Mohapatra, 2021; Table 1). In general, when plants are subjected to environmental stress, sRNA can promote plant adaptability *via* DNA methylation and maintain life activities.

**Table 1:** Examples of sRNA function.

Function	sRNA name	Targeted genes	Description	References
DNA Methylation	miR812w	*ACO3*, *CIPK10*, *LRR*	Overexpression of miR812w increased resistance to infection by the rice blast fungus *Magnaporthe oryzae*	Campo et al., 2021
	Mulberry 24 nt siRNA	*MET1*	24 nt siRNA reduced resistance gene methylation levels and increasing the plant’s resistance to *Botrytis cinerea*	Xin et al., 2021
	miR12477	*LAO*	Osa-miR12477 regulates *LAO* expression and alleviates oxidative damage in plant salt tolerance	Parmar et al., 2020
	TE-siR815	*WRKY45*	Te-sir815 induces transcriptional silencing of a key component of *WRKY45* signaling pathway through RdDM pathway, attenuate rice resistance to bacterial blight	Zhang et al., 2016a; Huang et al., 2019a
	miR165/166	*PHB*, *PHV*	Complementarity between *PHB* and *PHV* mRNA and miR165/166 is required for methylation of *PHB* and *PHV* genes	Song et al., 2019; Grzybkowska et al., 2020
RNA interference	miR396	*GRF4*, *GRF8*	*GRF4*, *GRF8* genes target site mutation, resulting in enlarged grain size and increased the BPH Resistance in their respective rice transgenic lines	Lin et al., 2021
	miR408	*PCY*	*PIF1* transmits external light signals to the interior Through miR408, and controls seed germination by converting *PCY* into internal hormonal profiles	Jiang et al., 2021
	miR160	*ARF10, ARF16, ARF17*	MiR160-*ARF*10/16/ 17 might serve as a molecular link in cross-talk of auxin, light, BR, and GA in hypocotyl elongation	Dai et al., 2021
	miR399	*PHO2*	MiR399-mediated *PHO2* regulation promote leaf stomatal development	Zhu et al., 2020b
	miR477	*CBP60a*	Ghr-miR477 directly cleaves the mRNA of *CBP60a*, regulates the biosynthesis of SA, and mediates plant defense	Hu et al., 2020
	siR109944	*TIR1*	Rice siR109944 suppresses plant immunity to sheath blight and impacts multiple agronomic traits by affecting auxin homeostasis	Qiao et al., 2020
	miR165/166	*REV*	In Arabidopsis, promotes AMs development through the miR165/166 target gene *REV*, giving rise to lateral shoots	Zhang et al., 2020
	miR845	Long terminal repeat	MiR845, targets the tRNA^Met^ PBS of LTR retrotransposons in *Arabidopsis* pollen, and triggers the accumulation of easiRNAs	Borges et al., 2018
Translation repression	miR172	*AP2*	MiR172 is important for flowering transition in many plants by inhibiting the expression level of translated *AP2*	Chen et al., 2018; Ó’Maoiléidigh et al., 2021
	miR156/miR159	*SPL9, MYB33*	Early in plant development, miR156 and miR159 inhibit the translation of *SPL9* and *MYB33*, respectively, and maintain the early nutritional stage	Fouracre et al., 2020
	Soybean 22 nt siRNA	*CHS*	Soybeans 22 nt siRNA targets the *CHS* gene to maintain the *CHS* mRNA content in the seed coat and keep the color of the soybean seed coat yellow	Jia et al., 2020
	*Arabidopsis* 22 nt siRNA	*NIA1*, *NIA2*	22 nt siRNA inhibits the translation of target genes and reduces the efficiency of protein conversion to cope with the stress of nitrogen deficiency	Wu et al., 2020
	miR858a	*MYBL2*	Overexpression of miR858a inhibits the expression of *MYBL2*, a key negative regulator of anthocyanin biosynthesis, enhances the accumulation of anthocyanins	Wang et al., 2016b

*ACO3: 1-AMINOCYCLOPROPANE-1-CARBOXYLIC ACID OXIDASE; CIPK10: CBL-INTERACTING PROTEIN KINASE10; LRR: LEUCINE-RICH REPEAT; MET1: MAKES METHIONINE 1; LOA: L-ASCORBATE OXIDASE; WRKY45: PHB: PHABULOSA; PHV: PHAVOLUTA; GRF: GROWTH REGULATING FACTOR; BPH: brown planthopper; PIF1: PHYTOCHROME INTERACTING FACTOR 1; PCY: PLANTACYANIN; ARF: AUXIN RESPONSE FACTOR; BR: brassinosteroid; GA: gibberellin; PHO2: PHOSPHATE2; CBP60a: Calmodulin-Binding Protein 60a; SA: salicylates; TIR1: TRANSPORT INHIBITOR RESPONSE 1; REV: REVOLUTA; AMs: Axillary meristems; PBS: primer-binding site; LTR: long terminal repeat; AP2: APETALA2; SPL9: SQUAMOSA PROMOTER BINDING PROTEIN-LIKE9; MYB33: MYB DOMAIN PROTEIN33; GHS: CHALCONE SYNTHASE; NIA: NITRATE REDUCTASE; MYBL2: MYB PROTO-ONCOGENE LIKE 2*.

One characteristic feature of sRNA functioning through RdDM is that parental DNA methylation markers can be maintained to the next generation without alteration of DNA sequence (Quadrana et al., 2016). For example, sRNA of *Trichoderma* spp. can participate in epigenetic regulation of plants through RdDM and induce immune response to protect plants (Morán-Diez et al., 2021). *Trichoderma atroviride* induces resistance to root-knot nematodes (RKN) in tomato, and importantly, the first generation of the tomato (F1) inherited resistance to RKN (Medeiros et al., 2017). After *Arabidopsis* is exposed to infection by biotrophic or necrotrophic pathogens, its progeny inherited resistance to biotrophic or necrotrophic pathogens across generations (López Sánchez et al., 2021). Trans-generational epigenetic inheritance of RdDM makes it possible to carry out plant genetic modification without changing the genotype and provides fundamental bases for the development of next-generation plant engineering approaches (Srikant and Drost, 2021).

Because of its role in DNA methylation-mediated transgenerational inheritance, sRNA can not only be applied to develop retro-resistant crops, but also to prevent triploid arrest, that is, to restore seed activity after hybridization of plants with different chromosome numbers. According to RdDM theory, 24 nt sRNA maintains TEs methylation. However, 21–22 nt easiRNA exists in *Arabidopsis* pollen, which is produced by miR845 targeting the tRNA^Met^ primer binding site (PBS) of the long terminal repeat (LTR) retrotransposon (Borges et al., 2018), this paternal easiRNA can prevent DNA methylation on TEs, leading to the overexpression of PEGs, failure of endosperm cellularization and seed abortion (Martinez et al., 2018). 22 nt easiRNA is generally increased in tetraploid pollen, so easiRNA is also considered to be a quantitative marker of paternal chromosome number (Martinez et al., 2018). Studies have shown that NRPD1a inhibits easiRNA formation and saves triploid seeds (Satyaki and Gehring, 2019). Nrpd1 inbreeding mutants have a continuously enhanced ability to inhibit triploid block due to the increased loss of DNA methylation at sites that are co-regulated by Chrome methylases 2 and 3 (CMT2/3), which further reflects the inter-generational inheritance of RdDM. Elucidation of the function of sRNA-mediated DNA methylation will benefit plant cultivation and agricultural production (Wang et al., 2021b).

### sRNA-Mediated Cleavage of Target mRNA–RNA Interference

sRNA-mediated target mRNA cleavage is also known as RNA interference (RNAi) in which sRNAs carried by RNA-induced silencing complex (RISC) are paired with target mRNAs according to the principle of base complementary pairing and the paired regions are cleaved with the participation of AGO protein (Figure 3B). This results in 5′ and 3′ end cleavages and post-transcriptional gene silencing for mRNA (Tan et al., 2020). sRNA-mediated target cleavage is widely used in the regulation of plant growth and development under normal conditions and plays an important role in abiotic and biotic stress response (Table 1). For example, MiR1885 in *Brassica* is naturally maintained at a low level, while it cleaves mRNA of R gene *BraTNL1* and keeps the R protein BraTNL1 at a controlled level to maintain basic immunity and nutritional development. After infection with *Turnip mosaic virus* (TuMV), HC-Pro protein suppressor blocks the miR1885-dependent inhibition of R gene and promotes the induction of *BraTNL1*, leading to the increased aggregation of immune receptors. Meanwhile, TuMV infection promotes the biosynthesis of miR1885 and triggers the synthesis of phasiR130-4 through the secondary siRNA biogenesis pathway (Figure 1E). Subsequently, phasiR130-4 mediates the silencing of photosynthesis-related gene *BraCP24*, leading to the acceleration of the floral transition and developmental defects in responding to viral infection (Cui et al., 2020). These examples also illustrate that one sRNA can target different sites, which complicates the regulatory network of sRNA.

As shown in Figure 2C, cross-kingdom RNAi of plant sRNA relies on EVs, which has been reported in plant- fungal pathogens interactions (Middleton et al., 2021). Plant-derived sRNA is contained in EVs and is easily absorbed by fungal cells (Cai et al., 2018). EVs in tomato root inhibit the spore germination and mycelia development of the plant pathogens *Fusarium oxysporum*, *Botrytis cinerea* and *Alternaria alternate*. Although studies have only focused on the protein cargo in EVs, we speculate that sRNA may play an important role in the suppression of tomato pathogens by EVs (De Palma et al., 2020). After EVs of sunflower are ingested by the fungal pathogens *Sclerotiania sclerotiorum*, the spores exhibit growth inhibition, morphological changes, and cell death (Regente et al., 2017). Studies have shown that many low-abundant sRNAs found in plants are abundant in fungi, and these sRNAs usually target genes important for infection to reduce the virulence of fungi (Cai et al., 2018; Thomma and Cook, 2018). When *Arabidopsis* is infected with *B. cinerea*, plant sRNAs, e.g., TAS1c-siR483 and TAS2-siR45, are delivered by EVs to cleave the mRNA of Bc-Vps51, Bc-DCTN1 and Bc-SAC1 in *B. cinerea*, leading to the silence of these target genes and reduced pathogenicity of *B. cinerea* (Cai et al., 2018). The targeting of the sRNA loaded in EV makes it possible to determine the key virulence factors of fungal pathogens by analyzing EV cargoes. However, it is still unknown how the EVs is accurately localized and how the fungus absorbs the EVs.

Fungal pathogens also deliver a series of sRNAs to plants to induce silencing of host immune genes. *B. cinerea* delivers Bc-siR3.1 to *Arabidopsis* and silences genes associated with oxidative stresses (Weiberg et al., 2013). The mechanism by which *Arabidopsis*-*B.cinerea* transmits sRNA to each other is designated as bidirectional cross-kingdom RNAi. This mechanism was also observed in cotton-*Verticillium* and wheat-*Fusarium graminearum* interactions (Zhang et al., 2016b; Jiao and Peng, 2018). This indicates that plant-fungal pathogens should be studied as an integral system. However, how to distinguish the origin of sRNA in this integral system has become a problem because both plants and fungal pathogens can produce sRNA. The genomes of most species have been sequenced, so the source of sRNA can usually be determined by homology search (Chen et al., 2021a). Using a sequential protoplast preparation method to purify fungal protoplasts from infected plant tissues, sRNAs that are transported from plant to fungal pathogen were identified (Cai et al., 2018). Fluorescent *in situ* hybridization can be used to study sRNA localization and expression (Huang et al., 2019b). The methods of fluorescein RNA label and fluorescent protein-sRNA vector construction are frequently used to study the absorption of sRNA (Wang et al., 2016a). The mechanisms by which EVs transport sRNA across the cell walls of plants and pathogens are still unknown. However, cell wall consists of interwoven fibrils and is incredibly elastic, which suggests that there is a potential way to control transport of sRNA by regulating the permeability of the cell wall (Coelho and Casadeval, 2019). Taken together, the bidirectional cross-kingdom RNAi mechanism requires to be further elucidated and discovered in more plant-fungal pathogens systems.

### sRNA-Mediated Translational Repression

sRNA needs to form RISCs with AGO1 to exert its translational repression function. Specifically, RISCs target the 3′ or 5′ untranslational region (UTR) of mRNA or the open reading frame (ORF) and inhibit translation by affecting ribosome movement and translation process in the endoplasmic reticulum (ER; Song et al., 2019; Figure 3C). While miRNA-mediated translational repression has been extensively reported (Table 1), there are relatively few studies on siRNA-mediated translational repression. Recently, 22 nt siRNA-mediated translational repression has been reported. Normally, protein *EIN5* and *SKI2* inhibit siRNA to avoid endogenous gene silencing (Zhang et al., 2015). When nitrogen nutrition in the environment is limited, two genes that encode nitrate reductases *NIA1* and *NIA2* in *Arabidopsis* produce large amount of 22 nt siRNA. While 22 nt siRNA does not reduce the transcription level of *NIA1* and *NIA2* genes, but it significantly inhibits the translation of mRNA, indicating that 22 nt targets the translation rather than transcription of *NIA1* and *NIA2*. This is a strategy for plants to adapt to the stress of nitrogen deficiency. Under the condition with limited nitrogen resources, the efficiency of protein translation and conversion is decreased, and the energy consumption is reduced in order to ensure the survival of the plants (Wu et al., 2020). In soybean, long inverted repeats (LIRs) located in the intron of a gene that is highly expressed in seed coat produces 22 nt siRNAs, which target the chalcone synthase (CHS) gene and trigger the biogenesis of secondary 21 nt siRNA. In the Gmdcl2a/2b mutant, 22 nt siRNAs and secondary 21 nt siRNAs cannot be produced, resulting in a significant increase in the accumulation of CHS mRNA in the seed coat, and changes of color of soybean seed coat from yellow to brown (Jia et al., 2020). sRNA can mediate translational inhibition, but the underlying mechanisms are still unclear, and need to be further explored (Ma et al., 2020). The sRNA-mediated translational inhibition has been widely used in various plant biological activities.

sRNA-mediated translational inhibition is also regulated by various proteins that are involved in the process of sRNA biogenesis. HYL1 is a member of D-body and mediates miRNA biosynthesis in the nucleus. However, recent studies have shown that *HYL1* exists in the cytoplasm and ER, and *HYL1* does not affect miRNA-mediated cleavage of target genes, but reduces the protein level of miRNA target genes by promoting translational repression (Yang et al., 2021a). Interestingly, it is generally believed that miRNA-mediated translational repression requires AMP1 gene, while siRNA-mediated translational repression does not require AMP1 gene (Wu et al., 2020; Yang et al., 2021a). However, recent studies have shown that AMP1 does not prevent translational repression of the SPL9 gene (target of miR156) or MYB33 gene (target of miR159), suggesting that AMP1 is not universally required for miRNA-mediated translational repression (Fouracre et al., 2020). In addition, sRNAs play an important role in the regulation of diverse plant phytohormones by controlling key factors involved in translational repression (Li et al., 2020). The plant hormone brassinosteroids (BRs) inhibit miRNA-mediated translational repression by negatively regulating the distribution of AGO1 in the ER of *Arabidopsis*. In BR-deficient mutants, the protein level of miRNA target genes is reduced, but can be recovered by BR treatment (Wang et al., 2021a). The controllability of sRNA-mediated translational inhibition suggests that it can be intervened manually, which provides novel strategies for the improvement of plant varieties.

## Conclusion and Outlook

Different types of sRNA are produced in the cell and reach the target site through different methods. Extensive studies on sRNA have formed a sRNA regulatory network. On one hand, based on this regulatory network, we can cultivate new traits of horticultural plants such as leaf development, flower development, fruit development and disease resistance by changing a certain process (Chen et al., 2018). On the other hand, we can actively promote population control and reduce the prevalence of plant diseases and insect pests through host-induced gene silencing (HIGS), nanoparticle-based exosome delivery of sRNA or spray-induced gene silencing (SIGS; Niu et al., 2021). However, there is still a long way to go before sRNA can be used in large-scale agriculture. New plant traits can be obtained by regulating sRNA’s ability to target mRNA, which requires more laboratory and field research in addition to the analysis of genomic results. The risks carried by genetically modified plants are also unpredictable. The study of plant EVs need to be rigorous and standardized (Pinedo et al., 2021), and the sRNA in a large number of plant EVs still need to be characterized. The application of SIGS needs to further optimize the stability of RNA in the environment and the delivery methods to improve the uptake efficiency by fungal pathogens (Qiao et al., 2021). It is highly expected that plant sRNA-based control strategies will be increasingly developed in the future to control plant diseases and insect pests and increase crop yields in an eco-friendly manner.

## Author Contributions

YT drafted the manuscript and the figures. XYa consulted the information on sRNA biogenesis and trafficking. CG consulted the information about the sRNA function. XYu conceived the idea and revised the manuscript. All authors contributed to the article and approved the submitted version.

## Funding

This study was supported by the National Natural Science Foundation of China (81872951 and 82173920), Natural Science Foundation of Zhejiang Province (LGN21H280002), and Zhejiang Xinmiao Talents Program (2021R410063).

## Conflict of Interest

The authors declare that the research was conducted in the absence of any commercial or financial relationships that could be construed as a potential conflict of interest.
